# Evaluation of anxiety levels and stress coping methods of pregnant women after the Kahramanmaraş earthquake

**DOI:** 10.3389/fpsyt.2026.1778774

**Published:** 2026-04-13

**Authors:** Sule Sirin Berk, Kemal Hansu, Oguz Akman

**Affiliations:** 1 Department of Obstetrics and Gynecology, Kahramanmaras Sutcu Imam Universitesi Tip Fakultesi, Kahramanmaras, Türkiye; 2 Department of Psychiatry, TC Sağlık bakanlığı Erdemli Devlet Hastanesi, Mersin, Türkiye

**Keywords:** adaptation, anxiety, earthquakes, pregnancy, psychological

## Abstract

**Objective:**

Natural disasters can cause serious psychological pressures on women during pregnancy. How the mental health of pregnant women is affected after major disasters such as earthquakes and what coping methods come into play in this process is an important research topic. This study aimed to evaluate the anxiety levels and stress coping strategies of pregnant women who experienced the February 6, 2023 Kahramanmaraş earthquake.

**Methods:**

This cross-sectional descriptive study was carried out within four months after the earthquake. A total of 118 pregnant women were included. Participants were grouped according to pregnancy trimester. Anxiety level was assessed with the Beck Anxiety Inventory and coping strategies with the Brief COPE Scale. Earthquake exposure data, including building damage and loss of relatives, were collected via structured survey.

**Results:**

The mean Beck Anxiety score was 15.9 ± 12.8. A significant difference was observed between trimesters (H = 19.09, p < 0.001), with anxiety declining from the first to the third trimester. Religious coping (ρ = 0.42, p < 0.001), acceptance (ρ = 0.36, p < 0.001), and behavioral avoidance (ρ = 0.36, p < 0.001) were positively correlated with anxiety. Positive reinterpretation and development showed a significant negative correlation with anxiety (ρ = −0.32, p < 0.001). Building damage category was not significantly associated with anxiety (p = 0.80).

**Conclusion:**

Anxiety in post-earthquake pregnant women differs according to trimester, and individual coping styles are associated with anxiety levels. Within the scope of the variables measured in this study, positive reinterpretation showed the strongest negative association with anxiety. Approaches supporting cognitive flexibility should be prioritized in perinatal mental health interventions.

## Introduction

Natural disasters put serious psychological pressure on women during pregnancy, leading to significant increases in the prevalence of perinatal mental disorders. Systematic reviews report that mental symptoms vary greatly depending on the type and severity of the disaster, for example, the prevalence of anxiety can be up to 48.2% in global crises such as pandemics (1). In studies conducted specifically for earthquakes, mental impact is mostly reported through depression and post-traumatic stress; In the study conducted approximately 8 months after the 2008 Sichuan earthquake, postpartum depression symptoms were detected in 29.0% of new mothers and post-traumatic stress disorder (PTSD) symptoms in 19.9%. It has been observed that these risks are much higher especially in women who are exposed to earthquakes at high levels (2). Similarly, post-earthquake studies conducted in Nepal revealed that the majority of mothers in the postnatal period, 72.4%, experienced moderate psychological stress (3). Other natural disasters such as hurricanes also support this picture; It is seen that the depression rates of pregnant and postpartum women exposed to Hurricane Katrina are similar to other pregnant and postpartum populations that have not experienced a disaster (4). Evidence from the February 6, 2023 Kahramanmaraş earthquake sequence further illustrates the breadth of these impacts: among 118 women surveyed in the aftermath, 38.1% exhibited PTSD symptoms, 77.8% reported difficulty sleeping, and 22.2% experienced post-earthquake violence, with financial hardship, housing damage, and anxiety identified as significant predictors of PTSD (5). In the light of these data, it can be said that approximately one-third of pregnant women are at risk of serious psychopathology, regardless of the type of disaster, and this situation constitutes an important public health problem for both maternal and fetal health (1).

Although the effects of natural disasters on maternal mental health are widely covered in the literature, there are significant gaps in post-disaster response strategies and pregnant women’s coping mechanisms. While most of the existing studies focus on the biological effects of disasters on fetal neurodevelopment or long-term risks, data on pregnant women’s access to health services and instant management of psychosocial difficulties they experience during crisis are insufficient (6). The number of evidence-based studies showing the effectiveness of individual education and care services, especially in disaster areas, in reducing pregnancy distress and birth anxiety is very limited (7). Coping strategy research in disaster-exposed pregnant populations remains particularly scarce. A notable exception is the QF2011 Queensland Flood Study, which examined 226 pregnant women exposed to severe flooding and found that emotion-focused coping was the most adaptive strategy under conditions of uncontrollable objective hardship, whereas both problem-focused and dysfunctional coping elevated subjective distress when exposure severity was high (8). In addition, it is seen that the special needs of pregnant women (logistics, mental health and medical support) are not systematically integrated in maternal crisis policies and this group is often neglected in emergency preparedness plans (9). Beyond individual coping resources, the role of social support in disaster recovery constitutes a critical yet underexplored dimension. Research has highlighted important distinctions between received social support, perceived social support, and social embeddedness, demonstrating that while communities often mobilize mutual helping in the immediate aftermath of disasters, a subsequent deterioration of perceived support and social cohesion can emerge over time and adversely affect survivors’ mental health (10). The complexity of post-disaster psychosocial problems in a sensitive period such as pregnancy makes the lack of multidisciplinary approaches and gap analyses more evident (11). As a result, there is an urgent need to determine specific coping strategies that will increase the post-disaster psychological resilience of pregnant women and support their adaptation processes for the next twenty years, when the frequency and severity of disasters are expected to increase (12).

The present study investigated both anxiety symptoms and stress coping methods among pregnant women who survived the February 6, 2023 Kahramanmaraş earthquake.The main hypothesis was that anxiety levels would differ according to pregnancy trimester and that specific coping strategies would exhibit significant associations with anxiety severity. In particular, it was predicted that adaptive strategies such as positive cognitive reframing would be associated with low anxiety, whereas avoidant and passive strategies would be associated with high anxiety levels. In addition, it is thought that the level of physical damage alone may not be the main factor determining psychological affectation and that the mental adjustment of pregnant women will be shaped more by individual coping resources. Accordingly, the study aimed to reveal the mental health profile of pregnant women in the disaster area, to determine trimester-specific anxiety patterns, and to define protective or risk-increasing coping mechanisms.

## Method

### Study population and sample

This cross-sectional analytical observational study was conducted between June and September 2023, following the February 6, 2023 Kahramanmaraş earthquakes. The inclusion criteria were: being over 18 years of age, having a pregnancy in any trimester, having experienced the February 6, 2023 earthquakes, being literate, and agreeing to participate in the study. Exclusion criteria were: a history of diagnosed psychiatric illness, presence of known chronic physical illness, hearing or vision loss that prevents communication, and inability to complete questionnaire forms.

The variables were recorded as follows. Age was taken as a whole year. Body mass index (BMI) was calculated by dividing body weight in kilograms by the square of height in meters (kg/m²). Educational status was evaluated in four categories: primary, secondary, high school, and university. Profession was grouped as housewife, teacher, and other. The teacher category was retained as a separate occupational group given the distinctly regulated working hours, institutional stress exposure, and psychosocial demands associated with this profession in the post-disaster context. Income level was classified as low, medium, and high according to the participants’ perceived economic status; this approach is a frequently used method in post-disaster populations when objective income verification is difficult (13).

The gestational period was categorized as first, second, and third trimesters (14, 15). Building damage was examined as undamaged, slightly damaged, moderately damaged, and heavily damaged or destroyed. Loss of relatives was evaluated in the categories of none, first degree, second degree, third degree, and friend. Smoking, alcohol, and substance use, and the presence of physical illness were questioned as yes/no, in line with previous studies examining psychosocial risk factors in post-disaster pregnant women (13). Being under the rubble was recorded as yes/no (15).

Earthquake exposure characteristics were also recorded. Participants were asked whether they were physically present in the earthquake zone at the time of the event, their location at the time of the earthquake, their post-earthquake housing or displacement status, and whether they were pregnant at the time of the earthquake. For participants who were pregnant at the time of the earthquake, gestational week and trimester at the time of the event were additionally recorded. Building damage status was assessed based on participant self-report.

Regarding the sampling procedure, participants were recruited consecutively from pregnant women attending the obstetrics outpatient clinic of Kahramanmaraş Sütçü İmam University Hospital during the data collection period. Of the women assessed for eligibility, those meeting all inclusion criteria and providing written informed consent were enrolled. Women who declined participation or did not meet the eligibility criteria were excluded.

### Power analysis

The sample size was calculated by taking into account the psychiatric morbidity rates and effect sizes reported in pregnant populations after natural disasters. Khatri et al. found that earthquakes caused antenatal diffuse mental disorders affecting 4.6% to 40.8% of pregnant women (15). Chen et al. showed that problem-focused coping strategies made a significant contribution to composite subjective distress in pregnant women exposed to the 2011 Queensland flood (β = +0.03, p = .02) (8). Gelaye et al. reported a moderate positive association between trauma exposure and anxiety scores among pregnant women (ρ = 0.39, p < 0.0001) (16). Based on these findings, a medium effect size of d ≈ 0.50 was assumed for the present study. The selection of a medium effect size was guided by Cohen’s (17) conventional criteria, which define d = 0.50 as a medium effect, and was further supported by the moderate associations reported in the disaster and perinatal mental health literature cited above (17). Using G*Power 3.1 software, a minimum sample of 118 participants was determined to be required to achieve 80% power at a 95% confidence level.

### Operating procedures

Data were collected by face-to-face interview. Participants were first administered a socio-demographic information form covering age, height, weight, education level, occupation, income level, gestational period, smoking/alcohol/substance use status, and presence of physical illness. Questions about earthquake experience were included in a separate section; these covered building damage status, experience of being under the rubble, loss of relatives, location at the time of the earthquake, direct earthquake exposure, post-earthquake displacement status, and gestational week and trimester at the time of the earthquake where applicable.

The Beck Anxiety Inventory was used to measure anxiety level. This scale consists of 21 items; each item is scored on a scale of 0–3, with a total score ranging from 0–63, where higher scores indicate greater anxiety. The Turkish validity and reliability study of the scale was conducted by Ulusoy et al. (18).

The Brief COPE Scale was used to evaluate coping strategies (19). The scale consists of 28 items and 14 subscales. The subscales are: use of useful social support, suppressing other preoccupations, restraint coping, planning, positive reinterpretation and development, coping with religion, humor, use of emotional social support, acceptance, behavioral avoidance, substance use, denial, mental letting go, and focusing on the problem and revealing emotions. Each subscale consists of two items and is scored in the range of 2–8. However, for the subscale of “focusing on the problem and revealing emotions, “ the total frequency score was calculated instead of the original two-item scoring, in order to evaluate the intensity of post-disaster stress responses more precisely; accordingly, the score range of this subscale differed from the standard 2–8 range. This composite structure and its scoring implications are described in detail in the relevant table notes. The Turkish adaptation of the scale was carried out by Bacanlı et al. (20).

The subscales were grouped under three coping types in accordance with the literature (8), following the classification model used by Chen et al. in post-disaster pregnancies (8). Consistent with Carver (19) and the Turkish adaptation by Bacanlı et al. (20), the subscales were classified as follows: problem-focused coping comprised use of useful social support, suppressing other preoccupations, restraint coping, and planning; emotion-focused coping comprised coping with religion, humor, use of emotional social support, acceptance, and positive reinterpretation and development; non-functional coping comprised behavioral avoidance, substance use, denial, mental letting go, and focusing on the problem and revealing emotions. The subscale “focusing on the problem and revealing emotions” was included in the non-functional coping group because it was considered a maladaptive strategy in the post-disaster context (8).

### Statistical analysis

Statistical analyses were performed using SPSS 25.0. Conformity of continuous variables to normal distribution was evaluated using the Shapiro–Wilk test. Non-parametric tests were preferred because the data were not normally distributed. Descriptive statistics were presented as mean ± SD for continuous variables with approximately normal distribution, and as median and interquartile range (IQR) along with minimum–maximum values for non-normally distributed continuous variables. In group comparisons, interquartile (25th and 75th percentile) values were reported along with the median. Categorical variables were expressed as frequency and percentage.

Spearman correlation analysis was applied to examine the relationship between Beck Anxiety scores and COPE subscales; the correlation coefficient is denoted as ρ throughout. The Kruskal–Wallis H test was used to compare three or more independent groups; this test was applied in comparisons by trimester and building damage category. For Kruskal–Wallis tests yielding a statistically significant result, pairwise *post-hoc* comparisons were conducted using Dunn’s test with Bonferroni correction; Bonferroni-corrected p-values (p-adj) are reported for each pairwise comparison (21). Effect sizes for Kruskal–Wallis comparisons were calculated using epsilon-squared (ϵ²), with values below 0.04 interpreted as negligible (17).

The statistical significance level was set at p < 0.05 and all analyses were performed two-tailed. Actual p-values were reported to two decimal places for p ≥ 0.01 and to three decimal places for p < 0.01; values below 0.001 were reported as p < 0.001. There were no missing data in the study.

### Ethical considerations

This study was approved by the Medical Research Ethics Committee of Kahramanmaraş Sütçü İmam University (Session No: 2025/31, Decision No: 03, Date: 10.11.2025). Written informed consent was obtained from all participants included in the study. In order to protect the privacy of the participants, personal data were anonymized, coded, and analyzed and used for research purposes only. The research process was carried out in accordance with the principles of the Declaration of Helsinki and full compliance with ethical principles was ensured.

The ethics committee approval for this study was obtained retrospectively. The data were collected between June and September 2023, in the immediate aftermath of the February 6, 2023 Kahramanmaraş earthquakes. Given the extraordinary nature of the disaster context, the urgent need to reach a highly vulnerable population, and the logistical constraints imposed by the crisis conditions, formal ethics committee application could not be completed prior to the initiation of data collection. The institutional ethics committee reviewed the study procedures and subsequently granted approval, recognizing the exceptional circumstances under which the research was conducted. Throughout the entire data collection process, all procedures were carried out in full accordance with the principles of the Declaration of Helsinki, including voluntary participation, written informed consent obtained from all participants, and strict protection of personal data through anonymization and coding.

## Results

### Characteristics of participants and earthquake experience

A total of 118 pregnant women were included in the study. The mean age was 26.6 ± 5.2 years and the mean body mass index was 26.6 ± 5.0 kg/m². When educational status was examined, high school graduates (n = 41, 34.7%) and university graduates (n = 34, 28.8%) constituted the largest groups among the participants. Housewives constituted the largest occupational group (n = 94, 79.7%). In terms of income level, the majority of participants reported a middle income level (n = 98, 83.1%). Regarding pregnancy periods, women in the third trimester constituted the largest group (n = 47, 39.8%), followed by the second (n = 38, 32.2%) and first trimesters (n = 33, 28.0%), respectively. No smokers, alcohol users, substance users, or participants with chronic physical illness were present in the study group. Regarding building damage, half of the participants resided in slightly damaged buildings (n = 59, 50.0%). Only one participant (0.8%) reported the experience of being under the rubble. Almost all participants reported losing a relative in the earthquake; loss of a friend was the most commonly reported type of loss (n = 51, 43.2%) (**Table 1A**).

**Table 1A:** Socio-demographic, clinical and earthquake exposure characteristics and beck anxiety score (n = 118).

Parameters	Value
Age (years)	26.6 ± 5.2
BMI (kg/m²)	26.6 ± 5.0
Education Status	
Primary School	10 (8.5%)
Secondary School	33 (28.0%)
High School	41 (34.7%)
University	34 (28.8%)
Profession	
Housewife	94 (79.7%)
Teacher	8 (6.8%)
Other	16 (13.6%)
Income Status	
Low	18 (15.3%)
Medium	98 (83.1%)
High	2 (1.7%)
Trimester at Time of Data Collection	
1st Trimester	33 (28.0%)
2nd Trimester	38 (32.2%)
3rd Trimester	47 (39.8%)
Smoking/Alcohol/Substance Use (None)	118 (100.0%)
Physical Illness (None)	118 (100.0%)
Building Damage	
Undamaged	28 (23.7%)
Slightly damaged	59 (50.0%)
Moderately damaged	22 (18.6%)
Heavily damaged + Destroyed	9 (7.6%)
Being Under the Rubble	
Yes	1 (0.8%)
No	117 (99.2%)
Loss of Relatives	
None	6 (5.1%)
1st Degree	12 (10.2%)
2nd Degree	29 (24.6%)
3rd Degree	20 (16.9%)
Friend	51 (43.2%)
Beck Anxiety Score	15.9 ± 12.8

Continuous variables are presented as mean ± standard deviation (SD); categorical variablesas n (%). The mean ± SD format was selected for continuous variables following confirmation of approximate normality via Shapiro–Wilk test; variables with non-normal distribution are presented as median (IQR) in **Table 4.** In the building damage variable, the severely damaged (n = 7) and destroyed (n = 2) groupswere combined due to small cell sizes. Building damage status was assessed based on participant self-report. Beck Anxiety Inventory score is presented here as a descriptive summary; group comparisons are presented in **Table 4**.

Regarding earthquake exposure characteristics, the majority of participants (n = 112, 94.9%)reported being directly present in the earthquake zone at the time of the event. The most common post-earthquake housing situation was temporary container or prefabricated housing (n = 38, 32.2%), followed by remaining in one’s own home (n = 31, 26.3%). Of the total sample, 44 participants (37.3%) were pregnant at the time of the earthquake; the median gestational age at the time of the earthquake in this subgroup was 10 weeks (IQR: 6–16). All pregnant participants at the time of the earthquake were in the first (n = 29, 65.9%) or second trimester (n = 15, 34.1%); no participant was in the third trimester at the time of the earthquake, as women who had reached the third trimester at that point would have already delivered prior to the data collection period. The remaining 74 participants (62.7%) became pregnant after the earthquake (**Table 1B**).

**Table 1B:** Earthquake exposure characteristics (n = 118).

Parameters	n	%
Location at Time of Earthquake		
Kahramanmaraş city centre	68	57.6%
Kahramanmaraş districts	32	27.1%
Other provinces	18	15.3%
Direct Earthquake Exposure		
Yes	112	94.9%
No	6	5.1%
Post-Earthquake Displacement Status		
Remained in own home	31	26.3%
Moved to relative’s or friend’s home	22	18.6%
Temporary container/prefabricated housing	38	32.2%
Tent city	18	15.3%
Other	9	7.6%
Pregnant at Time of Earthquake		
Yes	44	37.3%
No (became pregnant after earthquake)	74	62.7%
Gestational Week at Time of Earthquake (n = 44)	Median: 10 (IQR: 6–16)	—
Trimester at Time of Earthquake (n = 44)		
1st Trimester (0–12 weeks)	29	65.9%
2nd Trimester (13–26 weeks)	15	34.1%
3rd Trimester (27+ weeks)	0	0%

All data are based on participant self-report. Direct earthquake exposure was defined as being physically present in the earthquake zone at the time of the event. Displacement status reflects the living situation at the time of data collection (June–September 2023). No participant was in the third trimester at the time of the earthquake, as women who had reached the third trimester at that point (February 2023) would have already delivered prior to the data collection period.

### Relationships between coping and anxiety

The relationships between Beck Anxiety scores and COPE subscales were evaluated using Spearmancorrelation analysis. The strongest positive correlation with anxiety was observed in the religiouscoping subscale (ρ = 0.42, p < 0.001). Acceptance (ρ = 0.36, p < 0.001) and behavioral avoidance (ρ = 0.36, p < 0.001) also showed statistically significant positive correlations with anxiety. A weak but statistically significant positive correlation was found between use of emotional social support and anxiety (ρ = 0.20, p = 0.03). Positive reinterpretation and development showed a statistically significant negative correlation with anxiety (ρ = −0.32, p < 0.001). Focusing on the problem and revealing emotions was also negatively correlated with anxiety (ρ = −0.19, p = 0.04). No statistically significant correlation with anxiety was found for useful social support use (ρ = 0.10, p = 0.29), humor (ρ = 0.08, p = 0.37), restraint coping (ρ = 0.06, p = 0.54), mental letting go (ρ = 0.03, p = 0.75), substance use (ρ = 0.02, p = 0.80), planning (ρ = 0.00, p = 0.97), suppressing other preoccupations (ρ = −0.04, p = 0.66), or denial (ρ = −0.09, p = 0.34) (**Table 3**).

### Anxiety levels and coping strategies

The mean Beck Anxiety Inventory score of the participants was 15.9 ± 12.8. Among the COPEsubscales, the highest mean score was obtained for religious coping (6.1 ± 2.0), followed by positive reinterpretation and development (5.8 ± 1.7) and planning (5.7 ± 1.6). The lowest mean score was observed in substance use (3.2 ± 1.6). When coping type total scores were evaluated, emotion-focused coping had the highest mean (26.3 ± 4.8), followed by non-functional (24.1 ± 8.4) and problem-focused coping (21.7 ± 4.0) (**Table 2**).

**Table 2A:** COPE subscale scores (n = 118).

Subscale	BÇSÖ-KF factor	Item nos. (Bacanlı et al.,^20^)	Mean ± SD	Median [min–max]	Cronbach α (Bacanlı et al.,^20^)
Useful Social Support Use	ASDK	26, 15	5.4 ± 1.7	5 [2–8]	0.78
Suppressing Other Preoccupations	DEB	11, 17	5.1 ± 1.4	5 [2–8]	0.50
Restraint Coping	KS	28, 8	5.5 ± 1.5	5 [2–8]	0.39
Planning	PL	16, 22	5.7 ± 1.6	6 [2–8]	0.70
Positive Reinterpretation and Development	OYY	14, 21	5.8 ± 1.7	6 [2–8]	0.76
Coping with Religion	DİN	6, 27	6.1 ± 2.0	6 [2–8]	0.90
Humor	MİZ	7, 20	3.9 ± 1.6	4 [2–8]	0.92
Use of Emotional Social Support	DSDK	18, 9	5.4 ± 1.6	5 [2–8]	0.85
Acceptance	KAB	5, 25	5.1 ± 1.7	5 [2–8]	0.56
Behavioral Avoidance	DİK	4, 10	3.8 ± 1.8	4 [2–8]	0.59
Substance Use	MK	19, 12	3.2 ± 1.6	2 [2–8]	0.84
Denial	YAD	3, 23	4.1 ± 1.6	4 [2–8]	0.69
Mental Letting Go	ZİK	2, 24	4.7 ± 1.6	5 [2–8]	0.62
Focusing on the Problem and Revealing Emotions	DOK†	1, 13†	8.3 ± 7.2	6 [1–39]	0.70

Item numbers refer to the BÇSÖ-KF item numbering as reported in the Turkish adaptation (Bacanlı et al., 20). Each standard subscale consists of two items scored on a 1–4 Likert scale (subscale range: 2–8); no reverse scoring is applied to any subscale (Carver , 19; Bacanlı et al., 20). “Restraint Coping” (KS/Kendini Sınırlandırma) refers to waiting for the right opportunity before acting; this corresponds to the restraint coping construct in Carver (19) and Bacanlı et al. (20). Cronbach α values are those reported in Bacanlı et al. (20, 20), ranging from 0.39 (Restraint Coping) to 0.92 (Humor). ⚠ IMPORTANT NOTE: †The “Focusing on the Problem and Revealing Emotions” (DOK) subscale has a substantially different score range (1–39) from all other subscales (standard range: 2–8). This subscale was scored as a total frequency score across all applicable items rather than as a standard two-item subscale, as described in the Methods section. Direct comparison of raw scores between this subscale and others is not appropriate. BÇSÖ-KF: The short form of the Coping Styles Scale, USSU: ASDK, SOP: SOP, RC: RC, P: P, CR: DIN, H: MIZ, UESS: DSDK, A: A, PRD: PRD, BA: DIK, SU: MK, DAD: D, MLG: ZIK, FPRE: DOK.

**Table 2B:** Types of coping — total scores (n = 118).

Type of coping	Subscales included	Score range	Mean ± SD	Median [min–max]
Problem-focused	ASDK + DEB + KS + PL	8–32	21.7 ± 4.0	22 [8–32]
Emotion-focused	DİN + MİZ + DSDK + KAB + OYY	10–40	26.3 ± 4.8	26 [10–40]
Non-functional	DİK + MK + YAD + ZİK + DOK	10–40	24.1 ± 8.4	22 [10–54]

Coping category assignments follow Carver (19) and Bacanlı et al. (20). Problem-focused coping (4 subscales): Useful Social Support Use + Suppressing Other Preoccupations + Restraint Coping + Planning. Emotion-focused coping (5 subscales): Coping with Religion + Humor + Use of Emotional Social Support + Acceptance + Positive Reinterpretation and Development. Non-functional coping (5 subscales): Behavioral Avoidance + Substance Use + Denial + Mental Letting Go + Focusing on the Problem and Revealing Emotions. Positive Reinterpretation and Development (OYY) is classified as emotion-focused consistent with both Carver (19) and Bacanlı et al. (20). For full subscale-to-category mapping, see Table B. BÇSÖ-KF: The short form of the Coping Styles Scale, USSU: ASDK, SOP: SOP, RC: RC, P: P, CR: DIN, H: MIZ, UESS: DSDK, A: A, PRD: PRD, BA: DIK, SU: MK, DAD: D, MLG: ZIK, FPRE: DOK.

**Table 2C:** BÇSÖ-KF Subscale classification by coping category.

Subscale	BÇSÖ-KF factor	No. of items	Score range	Coping category (Carver, 19; Bacanlı et al., 20)	Source
Useful Social Support Use	ASDK	2	2–8	Problem-focused	Carver, 19; Bacanlı et al., 20
Suppressing Other Preoccupations	DEB	2	2–8	Problem-focused	Carver, 19; Bacanlı et al., 20
Restraint Coping	**KS**	2	2–8	Problem-focused	Carver, 19; Bacanlı et al., 20
Planning	PL	2	2–8	Problem-focused	Carver, 19; Bacanlı et al., 20
Coping with Religion	DİN	2	2–8	Emotion-focused	Carver, 19; Bacanlı et al., 20
Humor	MİZ	2	2–8	Emotion-focused	Carver, 19; Bacanlı et al., 20
Use of Emotional Social Support	DSDK	2	2–8	Emotion-focused	Carver, 19; Bacanlı et al., 20
Acceptance	KAB	2	2–8	Emotion-focused	Carver, 19; Bacanlı et al., 20 al., 2013
Positive Reinterpretation and Development	**OYY**	**2**	**2–8**	**Emotion-focused**	Carver, 19; Bacanlı et al., 20
Behavioral Avoidance	DİK	2	2–8	Non-functional	Carver, 19; Bacanlı et al., 20
Substance Use	MK	2	2–8	Non-functional	Carver, 19; Bacanlı et al., 20
Denial	YAD	2	2–8	Non-functional	Carver, 19; Bacanlı et al., 20
Mental Letting Go	ZİK	2	2–8	Non-functional	Carver, 19; Bacanlı et al., 20
Focusing on the Problem and Revealing Emotions	DOK‡	Composite	1–39‡	Non-functional	Bacanlı et al., 20

Coping category classifications are fully consistent with Carver (19) and the Turkish adaptation by Bacanlı et al., (20) “Restraint Coping” (KS/Kendini Sınırlandırma) refers to waiting for an appropriate opportunity to act, classified as problem-focused in the original framework (Carver, 19). “Positive Reinterpretation and Development” (OYY/Olumlu Yeniden Yorumlama) is classified as emotion-focused in both source documents; this classification has been applied consistently in the present study. Problem-focused strategies involve direct management of the stressor; emotion-focused strategies regulate emotional responses to stress; non-functional strategies involve avoidance-based responses that may hinder adaptive adjustment. ‡The standard BÇSÖ-KF DOK subscale comprises items 1 and 13 with range 2–8. The extended composite structure used in the present study (range 1–39) is described in the Methods section. BÇSÖ-KF: The short form of the Coping Styles Scale, USSU: ASDK, SOP: SOP, RC: RC, P: P, CR: DIN, H: MIZ, UESS: DSDK, A: A, PRD: PRD, BA: DIK, SU: MK, DAD: D, MLG: ZIK, FPRE: DOK.

**Table 3:** Association between beck anxiety and COPE subscales (spearman correlatioN, n = 118).

COPE subscale	Spearman ρ	p-value
Coping with Religion	0.42	< 0.001
Acceptance	0.36	< 0.001
Behavioral Avoidance	0.36	< 0.001
Use of Emotional Social Support	0.20	0.03
Useful Social Support Use	0.10	0.29
Humor	0.08	0.37
Restraint Coping	0.06	0.54
Mental Letting Go	0.03	0.75
Substance Use	0.02	0.80
Planning	0.00	0.97
Suppressing Other Preoccupations	−0.04	0.66
Denial	−0.09	0.34
Focusing on the Problem and Revealing Emotions	−0.19	0.04
Positive Reinterpretation and Development	−0.32	< 0.001

Spearman ρ = rank-order correlation coefficient. Statistically significant correlations (p < 0.05) are indicated in bold. Negative ρ values indicate an inverse association between the subscale score and anxiety level.

### Comparisons by trimester and building damage

Beck Anxiety scores differed significantly between trimester groups (H = 19.09, p < 0.001). The median anxiety score was highest in the first trimester (Median = 22, IQR: 15–30), followed by the second trimester (Median = 16, IQR: 9–24), and lowest in the third trimester (Median = 7, IQR: 4–12). Dunn’s *post-hoc* pairwise comparisons with Bonferroni correction indicated statistically significant differences between the first and third trimesters (p-adj < 0.001) and between the first and second trimesters (p-adj = 0.03). The difference between the second and third trimesters did not reach statistical significance after correction (p-adj = 0.08).

Useful social support use did not differ significantly between trimester groups(H = 0.10, p = 0.95). A statistically significant difference was found in the denialsubscale across trimesters (H = 6.88, p = 0.03); the lowest denial score was observed in the second trimester (3.5 ± 1.6). Dunn’s *post-hoc* analysis with Bonferroni correction revealed a statistically significant difference between the first and second trimester groups for denial (p-adj = 0.04); no significant pairwise differences were found for the remaining comparisons (Q1 vs Q3: p-adj = 0.99; Q2 vs Q3: p-adj = 0.07) (**Table 4A**).

**Table 4A:** Comparison by trimester groups (Kruskal–Wallis test with Dunn *Post-Hoc*, n = 118).

Parameters	Q1 (n = 33)	Q2 (n = 38)	Q3 (n = 47)	H (p)	Dunn *Post-Hoc* (Bonferroni-corrected p*)
Beck Anxiety (Median [IQR])	22 (15–30)	16 (9–24)	7 (4–12)	19.09 (< 0.001)*	Q1 vs Q2: p* = 0.03; Q1 vs Q3: p* < 0.001; Q2 vs Q3: p* = 0.08
Useful Social Support (Mean ± SD)	5.3 ± 1.6	5.5 ± 1.8	5.4 ± 1.8	0.10 (0.95)	Not applicable (overall H not significant)
Denial (Mean ± SD)	4.4 ± 1.3	3.5 ± 1.6	4.3 ± 1.8	6.88 (0.03)*	Q1 vs Q2: p* = 0.04; Q1 vs Q3: p* = 0.99; Q2 vs Q3: p* = 0.07

H, Kruskal–Wallis test statistic; IQR, Interquartile range (25th–75th percentile); *p < 0.05 considered statistically significant. p*, Bonferroni-corrected pairwise p-values from Dunn’s *post-hoc* test. The Q2 vs Q3 comparison for Beck Anxiety (p* = 0.08) did not reach statistical significance after Bonferroni correction.

**Figure 1:**
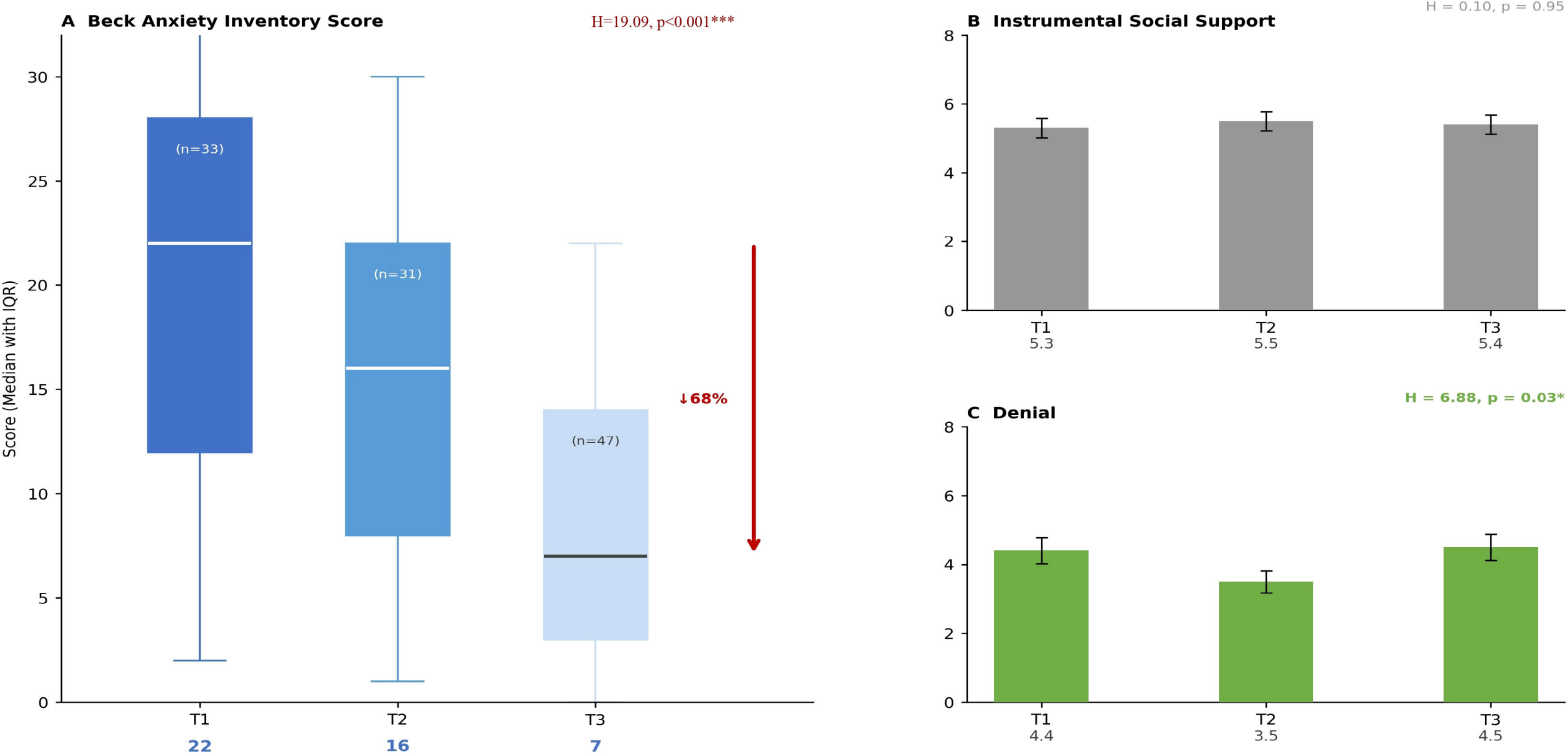
Beck anxiety and COPE subscales by trimester. *(A) Beck Anxiety scores; **(B)** Instrumental Social Support; **(C)** Denial. IQR, Interquartile Range; *p < 0.05, **p < 0.001.

In comparisons by building damage category, no statistically significant difference was found inBeck Anxiety scores (H = 1.03, p = 0.80), useful social support use (H = 3.13, p = 0.37), or the denial subscale (H = 4.10, p = 0.25). Effect sizes were negligible across all comparisons (ϵ² = 0.002–0.025). The heavily damaged and destroyed subgroup comprised only nine participants (n = 9, 7.6%), which substantially limits the statistical power of these comparisons; the absence of a statistically significant difference in this subgroup should therefore be interpreted with caution (**Table 4B**; **Figure 1**).

**Table 4B:** Comparison by building damage categories (Kruskal–Wallis test, n = 118).

Parameters	Undamaged (n = 28)	Slightly damaged (n = 59)	Moderately damaged (n = 22)	Heavy + destroyed (n = 9)	H (p)	Effect size (ϵ²)
Beck Anxiety (Median [IQR])	11 (4–20)	13 (6–27)	10 (6–30)	15 (8–18)	1.03 (0.80)	0.002
Useful Social Support (Mean ± SD)	5.8 ± 1.8	5.3 ± 1.7	5.0 ± 1.6	5.7 ± 2.2	3.13 (0.37)	0.018
Denial (Mean ± SD)	3.9 ± 1.4	4.1 ± 1.7	4.6 ± 1.8	3.6 ± 1.3	4.10 (0.25)	0.025

H, Kruskal–Wallis test statistic; IQR, Interquartile range; ϵ², Epsilon-squared effect size (negligible: < 0.04); all observed effect sizes were negligible (ϵ² = 0.002–0.025). No statistically significant difference was observed between building damage groups (all p > 0.05). The heavily damaged and destroyed subgroup comprised only nine participants (n = 9, 7.6%), which substantially limits the statistical power of these comparisons; findings should be interpreted with caution.

## Discussion

The research investigated how the February 6, 2023 Kahramanmaraş earthquake affected pregnancy-related anxiety in women and their methods for dealing with stress. Our findings point to several important points. The anxiety level of pregnant women remained below the values reported in the literature. Significant differences were observed according to the trimester of pregnancy; while early pregnant women exhibited higher anxiety, this level decreased in the following weeks. In terms of coping strategies, positive reinterpretation and development showed a negative correlation with anxiety, whereas strategies such as religious coping, acceptance, and behavioral avoidance were associated with high anxiety. The actual degree of building destruction was not aligned with the emotional impact experienced. The research indicates that pregnant women in the post-disaster period experience mental health changes based on their personal ability to cope and their social connections rather than their material losses.

In our study, the Beck Anxiety Inventory scores corresponded to mild-moderate anxiety. In the literature, higher anxiety scores are generally reported in pregnant populations after earthquakes (22). It is noteworthy that our findings fell short of these values. Although the majority of our sample reported bereavement, their anxiety level was lower than expected. However, the distribution of the type of loss may partially explain this situation: the most frequently reported loss was the loss of friends, while loss of first-degree relatives was comparatively rare. It is known that the loss of a first-degree relative affects the grieving process and related anxiety more deeply (22). The death of family members has been identified as one of the most important risk factors affecting mental health in women after disasters (23). In line with this, Samanci Tekin and Aydin (5) found that among 388 women surveyed after the Kahramanmaraş earthquake, 38.1% exhibited PTSD symptoms, and being single, having financial difficulties, and having a disaster-damaged home were significant predictors of distress — findings that underscore the multifactorial nature of post-earthquake psychopathology in women from this same context (5). Measurement timing can also be a factor influencing results. Our data were collected 1–4 months after the earthquake. The fact that the acute stress period had passed may have contributed to the decrease in anxiety scores. As a matter of fact, in a study conducted after the 1999 Marmara earthquake, evaluations in the first week pointed to higher stress levels (24). The fact that all our participants were healthy pregnant women who did not smoke, drink alcohol, or use substances can also be considered a moderating characteristic. The strong family structure and social support provided by kinship networks in Turkey seem to have been decisive in this finding. Similarly, Karagün et al. found that financial status or employment did not have a significant effect on anxiety and associated this with social support mechanisms (22). It is emphasized that women’s mental health is intertwined not only with psychological variables but also with social, cultural and spiritual factors (25). Research has highlighted that the benefits of social support following disasters depend critically on its perceived quality and continuity: while communities often mobilize mutual helping immediately after a disaster, this support can deteriorate over subsequent months as community resources are depleted, potentially undermining survivors’ mental health recovery (10). It is known that appropriate social support is vital for women in disaster areas and the risk of anxiety increases in its absence (23). In this context, the relatively low level of anxiety in our sample can be explained by the protective effect of cultural resilience and social support networks.

In our study, anxiety levels differed significantly between trimesters. The reduction from the first to the third trimester was quite remarkable and is in line with the observation that prenatal anxiety trajectories follow a dynamic and heterogeneous course (26). It has been shown that high stress levels in early gestational weeks significantly predict anxiety in the late period (26). High anxiety in the first trimester is probably due to the temporal proximity to the earthquake. Beyond temporal proximity, however, biological and obstetric factors likely contribute substantially to these differences. The first trimester is characterized by rapid hormonal fluctuations — particularly surges in progesterone and human chorionic gonadotropin (hCG) — that are independently associated with heightened emotional reactivity and anxiety (26). Concerns about miscarriage risk, which is highest in early pregnancy, and the absence of perceptible fetal movements may further amplify distress during this period. Previous pregnancy history, including prior pregnancy loss, complications, or infertility treatment, may also differentially sensitize women to anxiety in early gestation. It should be noted that the present study did not systematically collect data on these obstetric variables, including prior pregnancy complications, parity, or history of adverse pregnancy outcomes. This represents a methodological limitation that should be addressed in future studies, and the trimester-based findings should therefore be interpreted with this caveat in mind. It has been reported that pregnancy stress is significantly associated with both depression and anxiety trajectories (27). The decline in anxiety towards the third trimester can be explained by the introduction of adaptation mechanisms as time passes, and by the reassurance provided by feeling fetal movements. Psychological flexibility has been found to be associated with low anxiety in all pregnancy periods (26). The denial strategy also differed significantly according to trimester, with the lowest value observed in the second trimester — a period considered the most stable stage of pregnancy, during which there may be less need for cognitive defense mechanisms. It has been shown before that the effect of earthquake experience on pregnant women differs according to trimester (28), and that there is an increase in antenatal mental disorder symptoms in women who have experienced moderate-to-severe earthquakes (29). However, our data reveal that these symptoms may decrease over time.

In our study, religious coping strategy showed the strongest positive correlation with anxiety (ρ = 0.42, p < 0.001). This finding may seem surprising at first glance. It has been reported that religious practices are a protective mechanism that reduces perinatal anxiety and depression in Muslim women (30). However, the quality of religious coping seems to be decisive. In a study conducted in Iran, active religious coping was found to be inversely associated with anxiety, while passive and negative religious coping was directly proportional to anxiety (31). A similar situation may be present in our sample. Passive religious coping, which manifests itself as “consent to fate” and “surrender” in Turkish culture, may function as a reactive strategy used by individuals with high anxiety. In this context, Aka (32) proposes that spiritual and religious interventions targeting earthquake survivors should aim to modify dysfunctional and maladaptive perceptions through religious and spiritual resources — techniques such as reframing the disaster as a learning experience and strengthening hope — rather than reinforcing passive resignation (32). Acceptance (ρ = 0.36, p < 0.001) and behavioral avoidance (ρ = 0.36, p < 0.001) were also positively associated with anxiety. It has been previously determined that maladaptive strategies such as catastrophizing and rumination are positively correlated with anxiety in pregnant women (33). On the other hand, positive reinterpretation and development showed a significant negative correlation with anxiety (ρ = −0.32, p < 0.001), consistent with the literature. Bekircan et al. also reported a similar negative correlation between positive reinterpretation and negative post-traumatic cognition in the post-earthquake population (34), and it has been shown that post-traumatic negative cognitions increase as positive reinterpretation levels decrease (34). This pattern is further supported by the QF2011 Queensland Flood Study, in which emotion-focused coping — which includes positive reframing, acceptance, and humor — was the most adaptive strategy for pregnant women exposed to natural disaster, particularly under low-to-moderate levels of objective hardship; under high hardship conditions, emotion-focused coping also served as a buffer against subjective distress for women who frequently used maladaptive strategies (8). A noteworthy point is that although the total score of problem-focused coping was the highest among the three coping types, it did not show a significant relationship with anxiety. This finding parallels Chen et al.’s (8) observation that problem-focused coping was maladaptive under conditions of uncontrollable disaster-related hardship — a situation that closely resembles the earthquake context of the present study. This suggests that the quality and contextual appropriateness of the strategies are more decisive than the overall frequency of use. It is stated that mental health during pregnancy is shaped by the appropriate use of cognitive-emotional regulation strategies (33).

In our study, no statistically significant difference in anxiety scores was observed between building damage categories. However, this null finding should be interpreted with considerable caution. The heavily damaged and destroyed subgroup comprised only nine participants (n = 9, 7.6%), which results in very low statistical power for detecting even clinically meaningful differences in this group. The absence of a significant finding in these comparisons therefore reflects a substantially underpowered analysis rather than a conclusive demonstration of equivalence. The median anxiety scores of residents in undamaged and severely damaged buildings were numerically similar, yet the small sample prevents firm conclusions. In a study conducted after the earthquake in Japan, it was reported that psychological distress was significantly higher in areas with intense property damage (35). However, there is a different situation in our sample: the vast majority of participants had lost a loved one, which may have created a kind of ceiling effect in which social loss dominated as the primary stressor. Sudden and severe losses are known to have devastating effects on mental health (36), and it has been shown that social loss as a result of disasters leads to chronic grief and post-traumatic stress trajectories (37). In our data, social loss stands out as a dominant factor; the loss of physical property appears to have been overshadowed by this common trauma ground. It has been previously established that the nature of the loss, rather than external factors such as property loss, determines symptom trajectories (37). It is noteworthy that the use of useful social support was not significantly affected by building damage category. It is emphasized that social support plays a protective role in post-disaster mental health (38). Strong family and kinship systems in Turkish society may have acted as a buffer in this process. Although the 4-point difference between the undamaged and severely damaged groups was clinically plausible, the n = 9 severely damaged subgroup is far too small to detect this difference statistically; future studies with larger samples that include sufficient representation of severely exposed participants are needed to draw meaningful conclusions about the relationship between structural damage and anxiety in this population.

The research contains specific restrictions which affect its results. Due to the cross-sectional design, a causality relationship could not be established, and the findings only reflect the situation at the time of evaluation. The research results lack broad applicability because the study used data from a single specific location. The trimester-based comparisons are subject to two potential confounders that could not be disentangled in the present study: the time elapsed since the earthquake and the gestational age at which participants were exposed to the earthquake. Women in the first trimester at the time of data collection were necessarily closer in time to the earthquake and, if pregnant at the time of the disaster, were in earlier gestation than those in later trimesters. These factors may have independently contributed to the observed anxiety differences and cannot be separated from trimester effects in the current design. The small number of participants in the severely damaged building group (n = 9) substantially limited the statistical power of comparisons involving this subgroup, and these findings should not be interpreted as evidence of equivalence. Furthermore, the study did not collect data on several obstetric variables that may independently influence anxiety across trimesters, including hormonal profiles, parity, prior pregnancy loss, obstetric complications, and history of infertility treatment. The absence of these variables constitutes a methodological limitation, and future studies should systematically incorporate obstetric and reproductive history when examining trimester-based anxiety trajectories in disaster-exposed pregnant populations. The study also has notable strengths. Data were acquired at the beginning of the post-disaster recovery period, standardized assessment instruments were used, and direct interviews were conducted with earthquake survivors in the affected region. Future longitudinal studies should track the progression of pregnancy-related anxiety from early to late gestation in disaster-exposed populations. Studies evaluating religious coping quality across various cultural contexts would add value to the existing knowledge base. In particular, future research should examine whether spiritually sensitive interventions — such as those described by Aka (32) for earthquake survivors in Turkey — can reduce passive religious coping and strengthen adaptive emotion-focused strategies among pregnant women in disaster settings (32).

## Conclusion

The research findings showed that pregnant women in the earthquake disaster area developed mental health problems because of multiple factors which affected them. The extent of physical damage by itself does not determine the outcome, but pregnancy trimester and personal coping mechanisms of the individual become essential factors. Within the framework of the variables measured in this study, positive reinterpretation and development was the only coping strategy that showed a significant negative correlation with anxiety. However, other potential protective factors — including social support, cultural resilience, family structure, spiritual well-being, and obstetric history — were not systematically evaluated in this study, and their protective roles cannot be excluded. The research findings demonstrate that disaster zones require perinatal mental health services which not only address trauma but also incorporate methods that enhance cognitive flexibility. In particular, early psychosocial interventions targeting pregnant women in post-disaster settings should include structured cognitive reframing techniques — such as guided positive reappraisal exercises and cognitive restructuring approaches — that directly strengthen the capacity for adaptive emotion-focused coping. Emergency response plans should include specialized support systems which protect pregnant women who are particularly vulnerable during disaster emergencies.

## Data Availability

The original contributions presented in the study are included in the article/supplementary material.Further inquiries can be directed to the corresponding author.
